# Altered Functional Connectivity of Amygdala Subregions with Large-Scale Brain Networks in Schizophrenia: A Resting-State fMRI Study

**DOI:** 10.3390/tomography12010002

**Published:** 2025-12-23

**Authors:** Rasha Rudaid Alharthi, Duaa Banaja, Adnan Alahmadi, Jaber Hussain Alsalah, Arwa Baeshen, Ali H. Alghamdi, Magbool Alelyani, Njoud Aldusary

**Affiliations:** 1 Department of Radiologic Sciences, Faculty of Applied Medical Sciences, King Abdulaziz University, Jeddah 21589, Saudi Arabia; ralharthi0229@stu.kau.edu.sa (R.R.A.); dbanaja@kau.edu.sa (D.B.); aaalahmadi@kau.edu.sa (A.A.); jhalyami@kau.edu.sa (J.H.A.); 2 Smart Medical Imaging Research Group, King Abdulaziz University, Jeddah 21589, Saudi Arabia; 3 King Fahd Medical Research Center, King Abdulaziz University, Jeddah 21589, Saudi Arabia; 4 Radiological Sciences Department, College of Applied Medical Sciences, King Saud University, Riyadh 11433, Saudi Arabia; abaeshen@ksu.edu.sa; 5 Department of Radiological Sciences, Faculty of Applied Medical Sciences, University of Tabuk, Tabuk 71491, Saudi Arabia; ah.alghamdi@ut.edu.sa; 6 Department of Radiological Sciences, College of Applied Medical Sciences, King Khalid University, Abha 61421, Saudi Arabia; maalalyani@kku.edu.sa

**Keywords:** schizophrenia, healthy control subjects, centro-medial amygdala, latero-basal amygdala, functional connectivity, fMRI, brain networks

## Abstract

**Objective:** This study aimed to investigate the functional connectivity (FC) of three amygdala subregions—the laterobasal amygdala (LBA), centromedial amygdala (CMA), and superficial amygdala (SFA)—with large-scale brain networks in individuals with schizophrenia (SCZ) compared to healthy controls (HC). **Methodology:** Resting-state functional magnetic resonance imaging (rs-fMRI) data were obtained from 100 participants (50 SCZ, 50 HC) with balanced age and gender distributions. FC between amygdala subregions and target functional networks was assessed using a region-of-interest (ROI)-to-ROI approach implemented in the CONN toolbox. **Result:** Connectivity patterns of the LBA, CMA, and SFA differed between SCZ and HC groups. After false discovery rate (FDR) correction (*p* < 0.05), SCZ patients exhibited significantly increased FC between the left CMA and both the default mode network (DMN) and the visual network (VN). In contrast, decreased FC was observed between the right LBA and the sensorimotor network (SMN) in SCZ compared with HC. **Conclusions:** These findings reveal novel FC alterations linking amygdala subregions with large-scale networks in schizophrenia. The results underscore the importance of examining the amygdala as distinct functional subregions rather than as a single structure, offering new insights into the neural mechanisms underlying SCZ.

## Simple Summary

Schizophrenia is a known disorder cause disruptions in functional connectivity. A crucial element in the pathophysiology of schizophrenia, the amygdala is composed of various subregions that may have different effects on the disorder. Few studies have addressed the amygdala subregions, and none have specifically examined the amygdala subregion connectivity to large-scale resting-state networks, leaving a critical gap in our understanding of how subregion-specific amygdala dysfunction integrates with systems-level network pathology in schizophrenia. By examining these subregions, we gained a better understanding of the neural mechanisms underlying schizophrenia. These connectivity patterns will help develop interventions aimed at restoring balanced amygdala–network interactions.

## 1. Introduction

Schizophrenia (SCZ) is a severe psychiatric disorder marked by disturbances in emotion, cognition, and behavior that compromise social functioning and quality of life [1]. Globally, approximately 24 million individuals live with SCZ [2]. Clinical presentation spans positive symptoms (e.g., hallucinations, delusions), negative symptoms (e.g., avolition, social withdrawal), and cognitive deficits in working memory, executive functioning, and processing speed, each contributing substantially to long-term disability and poorer outcomes [3,4]. Diagnosis is established using DSM-5 criteria [5]. SCZ typically emerges in late adolescence or early adulthood—with earlier onset in males—and is associated with widespread brain alterations and a substantial burden, including elevated suicide risk [2,6,7].

Functional magnetic resonance imaging (fMRI) offers a powerful, noninvasive window into the neural systems implicated in SCZ. Blood-oxygenation-level–dependent (BOLD) fMRI indexes hemodynamic responses to neuronal activity [8]. Resting-state fMRI (rs-fMRI), which examines spontaneous low-frequency BOLD fluctuations in the absence of tasks [9,10,11], has been widely adopted in psychiatry and neurology due to its feasibility across clinical populations. Analytic approaches to rs-fMRI commonly quantify functional connectivity (FC)—the temporal coupling of BOLD signals across regions—as a systems-level marker of brain organization [8,12]. Consistent networks reproducibly emerge at rest, including the default mode (DMN), sensorimotor (SMN), visual (VN), salience (SN), dorsal attention (DAN), frontoparietal (FPN), language (LN), and cerebellar (CN) networks [10,13,14,15,16,17,18].

Converging evidence supports a “disconnection” framework in SCZ, positing that aberrant large-scale network coupling underlies core symptoms and cognitive deficits [19,20,21,22,23]. Meta-analyses and empirical studies report altered FC within and between multiple networks—including DMN, FPN, SN, SMN, and VN—suggesting widespread systems-level dysfunction [19,20,24,25,26].

Within this system’s context, the amygdala is a central limbic hub for affect, salience, and socioemotional processing and has long been implicated in SCZ [27,28,29]. Crucially, the amygdala is not unitary. Human cytoarchitectonic and connectivity-based parcellations consistently identify three subregions—the laterobasal (LBA), centromedial (CMA), and superficial (SFA)—with distinct afferent–efferent profiles and functions [30,31,32,33,34]. Broadly, LBA shows strong coupling with temporal–limbic and medial prefrontal cortices; CMA interfaces with motor, thalamic, cerebellar, and striatal systems; and SFA is linked to limbic and cingulate territories [35]. Structural studies in SCZ report subregion-specific abnormalities, including volume reductions in accessory basal nuclei and greater left LBA/CMA atrophy relative to bipolar disorder [36,37,38]. Yet, despite this anatomical differentiation, most FC studies have treated the amygdala as a single region.

Only a small number of rs-fMRI investigations have examined amygdala subregion FC in SCZ. Liu et al. (2014) reported altered coupling between amygdala subregions (CMA, LBA, SFA) and prefrontal ROIs, while Zhang et al. (2020) found increased FC between right CMA and postcentral gyrus and decreased FC between right LBA and precentral gyrus in first-episode SCZ. However, these studies did not systematically map amygdala subregion connectivity to large-scale resting-state networks, leaving a critical gap in our understanding of how subregion-specific amygdala dysfunction integrates with systems-level network pathology in SCZ [21,39].

The present study addresses this gap by characterizing FC within and between amygdala subregions (LBA, CMA, SFA) and by mapping their connectivity with eight resting-state networks (DMN, SN, FPN, DAN, SMN, VN, LN, CN) in SCZ versus healthy controls (HC). We hypothesized that SCZ would show (i) preserved but strength-altered intra-amygdala FC; (ii) hyperconnectivity between limbic/sensory networks and amygdala subregions (e.g., CMA–DMN, amygdala–VN/LN); and (iii) hypoconnectivity between amygdala subregions and control networks (e.g., FPN, SN, SMN). By resolving amygdala circuitry at the subregional level within a whole-brain network framework, this work aims to refine mechanistic models of SCZ and inform the development of subregion- and network-targeted biomarkers and interventions.

## 2. Materials and Methods

Data were obtained from the OpenfMRI database (DS000030) as part of the Consortium for Neuropsychiatric Phenomics dataset [40], which is publicly available and free of usage restrictions. The sample comprised 100 adults aged 22 to 50 years, including 50 patients diagnosed with schizophrenia (SCZ) and 50 healthy controls (HC). Groups were balanced for sex, with 38 men and 12 women in each group.

Participants were eligible if they self-identified as either White, Not of Hispanic or Latino Origin, or Hispanic or Latino of any race, according to the U.S. National Institutes of Health racial/ethnic categories, and had completed at least eight years of formal education. All participants underwent resting-state functional magnetic resonance imaging (rs-fMRI) and comprehensive neuropsychological testing. Exclusion criteria included a history of neurological disease, head injury, current use of psychoactive medications, recent substance dependence, or the presence of major psychiatric disorders other than schizophrenia, attention-deficit/hyperactivity disorder, or current mood or anxiety disorders. Psychiatric histories were verified with the Structured Clinical Interview for DSM-IV (SCID-IV), and urine toxicology screening was performed to exclude individuals testing positive for cannabis, amphetamines, opioids, cocaine, or benzodiazepines. Additional exclusions for MRI acquisition included left-handedness, suspected pregnancy, and contraindications such as claustrophobia, metal implants, or body size incompatible with the scanner bore. During rs-fMRI scanning, participants were instructed to remain awake with eyes open and to relax for five minutes without performing any specific task.

All imaging was conducted on a Siemens Trio 3T scanner (Siemens Healthineers, Erlangen, Germany). Functional images were acquired using a T2*-weighted echo-planar imaging (EPI) sequence with the following parameters: repetition time (TR) = 2 s, echo time (TE) = 30 ms, flip angle = 90°, matrix size = 64 × 64, field of view (FOV) = 192 mm, slice thickness = 4 mm, and 34 axial slices. High-resolution structural images were collected using a T1-weighted MPRAGE sequence (TR = 1.9 s, TE = 2.26 ms, FOV = 250 mm, matrix size = 256 × 256, slice thickness = 1 mm, 176 slices).

Preprocessing of the fMRI data was performed using the CONN toolbox (version 22a, NITRC, Cambridge, MA, USA; https://web.conn-toolbox.org/) in conjunction with Statistical Parametric Mapping software (SPM12, Wellcome Centre for Human Neuroimaging, London, UK; https://www.fil.ion.ucl.ac.uk/spm/, access on 15 October 2023). Standard procedures included slice-timing correction, realignment of functional volumes, and normalization to the Montreal Neurological Institute (MNI) template using individual structural images. CONN’s default motion thresholds were used to identify artifacts using Artifact Detection Tools (ART). Outlier volumes were recognized when they exceeded a global BOLD signal change in five standard deviations (SD) or a framewise displacement (FD) of 0.9 mm. To effectively eliminate their impact, these outlier volumes were included as nuisance regressors. Functional volumes were spatially smoothed with an 8 mm full-width at half-maximum Gaussian kernel, because this standard group-level smoothing size enhances the signal-to-noise ratio and accommodates inter-subject anatomical heterogeneity. Temporal preprocessing involved denoising to reduce the influence of confounding signals from white matter, cerebrospinal fluid, head motion, and scrubbing regressors on the blood-oxygen-level–dependent (BOLD) signal.

**Figure 1:**
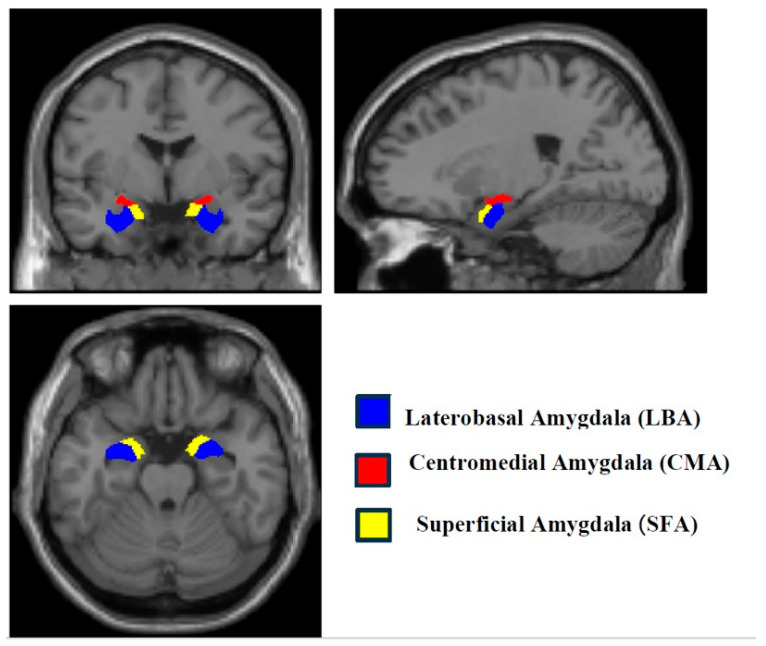
Illustration of amygdala subregions (LBA, CMA, SFA).

Regions of interest (ROIs) included three amygdala subregions in each hemisphere—Laterobasal (LBA), Centromedial (CMA), and Superficial (SFA)—defined using the SPM Anatomy Toolbox (Institute of Neuroscience and Medicine, Forschungszentrum Jülich, Jülich, Germany) (Figure 1). In addition to the subregion-based parcellation, an auxiliary analysis was conducted using right and left whole amygdala as ROIs to complement and contextualize the subregion-level findings. Functional networks of interest, as specified in the CONN atlas, comprised the default mode (DMN), salience (SN), frontoparietal (FPN), dorsal attention (DAN), sensorimotor (SMN), visual (VN), cerebellar, and language (LN) networks.

Functional connectivity was analyzed at two levels. At the first (individual) level, Fisher-transformed bivariate correlation coefficients were calculated between the time series of each pair of ROIs using weighted general linear models to generate subject-specific connectivity matrices. At the second (group) level, ROI-to-ROI analyses were performed to compare connectivity patterns between SCZ and HC groups. Group differences were assessed using two-sample *t*-tests and F-tests. All ROI-to-ROI comparisons were combined into CONN’s second-level model and each ROI-to-ROI connection was tested independently to address the correlation between amygdala subregions and prevent inflation of significance. Multiple comparisons were then corrected using ROI-level false discovery rate (FDR) correction at *p* < 0.05. This method controls the expected percentage of false positives while taking into account the interdependence among ROIs. Cluster-level inferences based on multivariate parametric statistics [41]. FDR correction was preferred over family-wise error correction for its greater sensitivity in detecting distributed network effects [42,43].

## 3. Results

### 3.1. Functional Connectivity Within Amygdala Subregions

**Figure 2:**
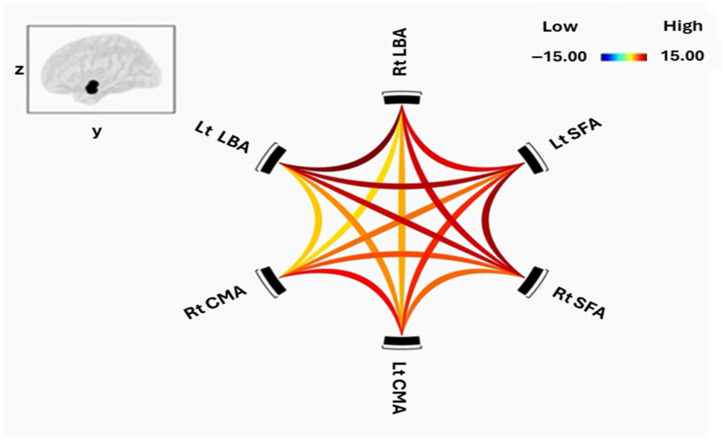
Functional connectivity within amygdala subregions of HC. The cluster threshold was set at *p* < 0.05 p-FDR corrected, and the connection threshold was set at *p* < 0.05 p-uncorrected.

**Figure 3:**
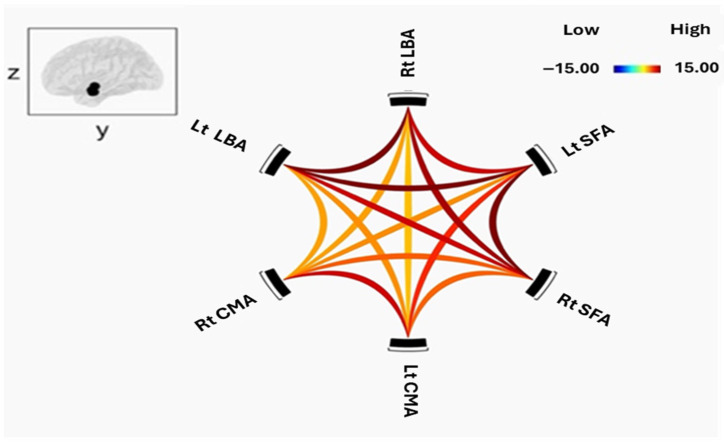
Functional connectivity within amygdala subregions of SCZ. The cluster threshold was set at *p* < 0.05 p-FDR corrected, and the connection threshold was set at *p* < 0.05 p-uncorrected.

All three amygdala subregions—the laterobasal (LBA), centromedial (CMA), and figusuperficial (SFA) nuclei demonstrated significant positive resting-state functional connectivity (FC) within and across hemispheres in both schizophrenia (SCZ) patients and healthy controls (HC). Figure 2 and Figure 3 illustrate intra-amygdala connectivity patterns and the strength of individual connections for HC and SCZ groups, respectively.

In the SCZ group, the right LBA showed robust connectivity with the left LBA and bilateral SFA, but comparatively weaker connectivity with bilateral CMA. The left LBA exhibited a similar pattern, with strong connectivity to bilateral SFA and weaker connections to bilateral CMA, confirming a preserved intrinsic connectivity architecture in patients. The right CMA was strongly coupled with the left CMA and right SFA, with weaker connections to the left SFA and bilateral LBA. The left CMA also has strong connections with the right CMA and bilateral SFA. Both right and left SFA demonstrated strong connectivity with all other subregions, although the left SFA exhibited weaker coupling with the right CMA.

Healthy controls showed a broadly similar pattern of positive intra-amygdala connectivity, including strong bilateral LBA coupling and robust LBA–SFA connections. CMA–SFA connectivity was also strong, though CMA–LBA connections were comparatively weaker.

Between-group comparison using two-sample *t*-tests revealed no significant differences in FC strength within or between amygdala subregions after false discovery rate (FDR) correction.

### 3.2. Connectivity Between Amygdala Subregions and Large-Scale Brain Networks

**Figure 4:**
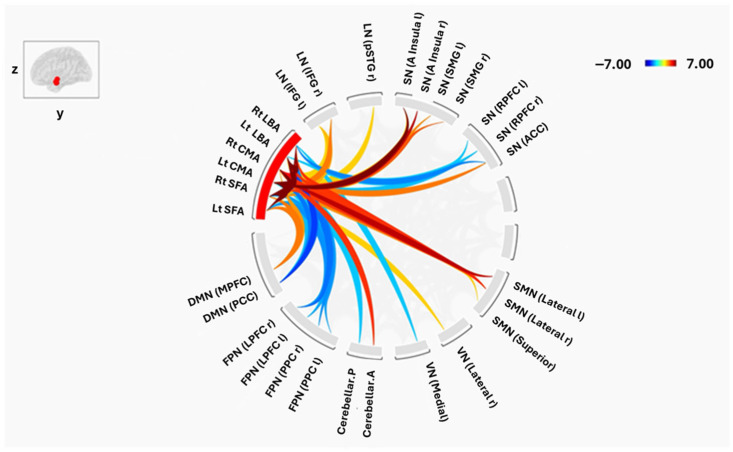
Functional connectivity between amygdala subregions and Brain Networks in HC. The cluster threshold was set at *p* < 0.05 p-FDR corrected, and the connection threshold was set at *p* < 0.05 p-uncorrected. The red and blue lines indicate positive and negative connectivity, respectively.

**Figure 5:**
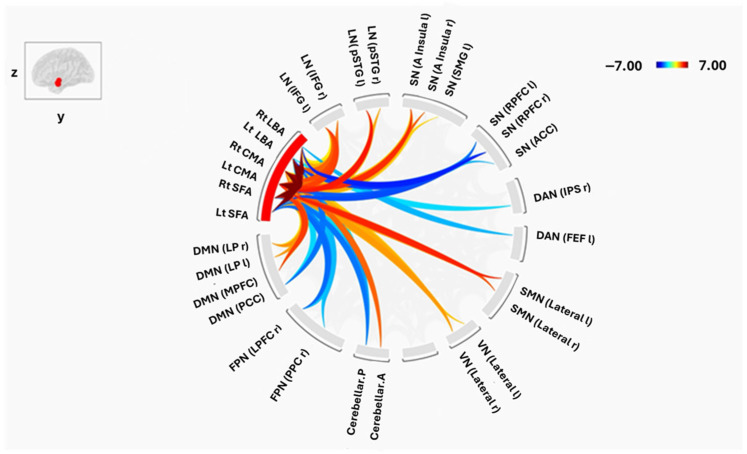
Functional connectivity between amygdala subregions and Brain Networks in SCZ. The cluster threshold was set at *p* < 0.05 p-FDR corrected, and the connection threshold was set at *p* < 0.05 p-uncorrected. The red and blue lines indicate positive and negative connectivity, respectively.

Despite similar intra-amygdala patterns, group differences emerged in the connectivity of amygdala subregions with distributed resting-state networks (Figure 4 and Figure 5). Both positive and negative FC were observed across default mode (DMN), salience (SN), sensorimotor (SMN), visual (VN), frontoparietal (FPN), language (LN), dorsal attention (DAN), and cerebellar (CN) networks.

#### 3.2.1. Laterobasal Amygdala (LBA)

In HCs, the right LBA showed significant positive FC with the bilateral lateral SMN and the superior SMN, whereas SCZ patients demonstrated positive connectivity with bilateral inferior frontal gyrus (IFG) of the LN and the right lateral VN. Negative FC in HCs involved the lateral prefrontal cortex (LPFC), posterior parietal cortex (PPC) of the FPN, anterior cingulate cortex (ACC) of the SN, and PCC of the DMN (all *p* < 0.01). SCZ patients also exhibited negative FC with the rostral PFC, ACC, frontal eye field of the DAN, posterior CN, and PCC of the DMN.

The left LBA in HCs displayed positive FC with bilateral lateral SMN, left, and left insula, whereas SCZ patients showed positive FC with bilateral IFG, left insula, and right lateral VN. Negative FC in HCs encompassed the PCC of the DMN, bilateral LPFC and PPC of the FPN, and posterior CN (all *p* < 0.01), while SCZ patients showed negative FC with similar regions, including the PCC and right PPC.

#### 3.2.2. Centromedial Amygdala (CMA)

Both groups exhibited positive FC of the right CMA with bilateral SMN, bilateral insula of the SN, anterior CN, right posterior superior temporal gyrus (pSTG), and right IFG of the LN (all *p* < 0.05). However, HCs additionally showed positive FC with supramarginal gyrus (SMG) of the SN and MPFC of the DMN, whereas SCZ patients demonstrated positive FC with the MPFC, bilateral lateral parietal DMN nodes, and left pSTG. Negative FC was absent in SCZ but present in HCs, including the PCC of the DMN, bilateral PPC, and right LPFC of the FPN.

The left CMA exhibited a comparable pattern: both groups showed strong FC with SMN, insula, anterior CN, and bilateral IFG. HCs demonstrated additional positive FC with bilateral SMG and ACC of the SN, whereas SCZ patients showed unique positive FC with the MPFC, bilateral lateral parietal DMN nodes, and left pSTG. Negative FC was again broader in HCs, involving PCC, PPC, and LPFC.

#### 3.2.3. Superficial Amygdala (SFA)

In HCs, the right SFA displayed positive FC with bilateral lateral SMN, MPFC of the DMN, bilateral insula of the SN, right SMG, right pSTG of the LN, and right lateral VN (all *p* < 0.05), while negative FC was observed with bilateral PPC and LPFC of the FPN and posterior CN. SCZ patients exhibited positive FC with MPFC, right IFG, bilateral lateral SMN, bilateral lateral VN, right LP of the DMN, and right pSTG of the LN, and negative FC with salience rostral PFC, right LPFC of the FPN, posterior CN, and PPC.

The left SFA in HCs showed positive FC with MPFC, bilateral lateral SMN, bilateral IFG, and left insula, whereas SCZ patients displayed positive FC with bilateral IFG and pSTG, MPFC, left insula, right lateral VN, and left SMG. Negative FC in HCs included bilateral LPFC and PPC of the FPN, PCC of the DMN, and salience rostral PFC, while SCZ patients demonstrated negative FC with salience rostral PFC, right LPFC, posterior CN, and PCC.

### 3.3. Group-Level Differences

**Figure 6:**
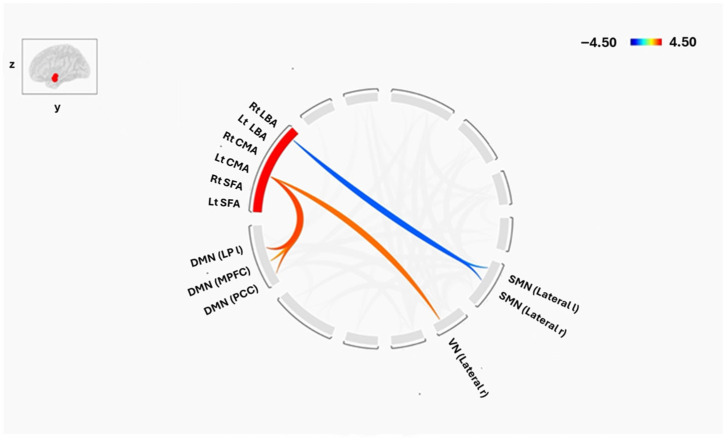
Functional Connectivity Differences in amygdala subregions and Brain Networks, between Groups (SCZ and HC). The red color shows the higher connectivity in SCZ compared to HC as a direct comparison, while the blue is vice versa. The cluster threshold was set at *p* < 0.05 p-FDR corrected, and the connection threshold was set at *p* < 0.05 p-uncorrected.

Direct group comparisons (two-sample *t*-tests, cluster-level *p* < 0.05 FDR-corrected) revealed the most significant differences between groups, establishing the core findings of this study. Specifically, higher FC in SCZ between the left CMA and several key DMN nodes, including the left lateral parietal cortex (*p* = 0.01), MPFC (*p* = 0.03), and PCC (*p* = 0.01), as well as with the right lateral VN (*p* = 0.02). In contrast, higher FC in HCs was observed between the right LBA and bilateral lateral SMN (right: *p* = 0.01; left: *p* = 0.02), indicating a relative reduction in the connectivity between LBA and SMN in the SCZ group. These effects are summarized in Figure 6.

**Figure 7:**
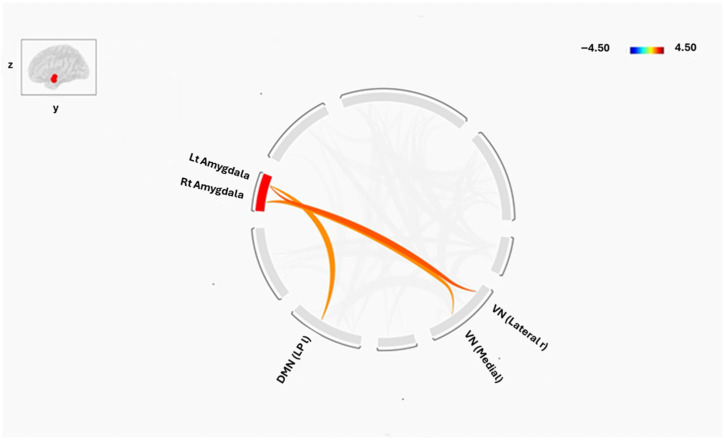
Functional Connectivity Differences in bilateral amygdala and Brain Networks between Groups (SCZ and HC). The red color shows the higher connectivity in SCZ compared to HC as a direct comparison, while the blue is vice versa. The cluster threshold was set at *p* < 0.05 p-FDR corrected, and the connection threshold was set at *p* < 0.05 p-uncorrected.

To strengthen the rationale for subregion-specific analysis, an additional whole-amygdala analysis was performed. When treating the amygdala as a single bilateral structure showed higher FC in SCZ between the right amygdala and the medial (*p* = 0.04) visual networks compared to healthy controls. The left amygdala, in contrast, revealed an increase in FC with the right lateral visual network (*p* = 0.03) and the left lateral parietal cortex of DMN (*p* = 0.04). These connections are summarized in Figure 7, where red lines denote stronger connectivity in SCZ.

## 4. Discussion

This study investigated rs-FC patterns of the amygdala subregions—LBA, CMA, and SFA—and their large-scale network associations in patients with SCZ compared with HCs. By examining these subregions separately, we aimed to provide a better understanding of the neural mechanisms underlying SCZ, a disorder characterized by profound emotional, cognitive, and behavioral disturbances that disrupt social functioning and reality perception.

Neuroimaging research increasingly highlights the importance of studying amygdala subregions as distinct functional units. Recent studies have demonstrated subregion-specific FC alterations in a range of neuropsychiatric conditions, including autism [44], anxiety [45], obsessive–compulsive disorder [46], post-traumatic stress disorder [47], and major depressive disorder [48]. These findings underscore that the LBA, CMA, and SFA should not be treated as a single homogeneous region but rather as functionally differentiated nuclei with unique contributions to emotional processing and regulation.

Structural MRI studies have also reported amygdala subregional abnormalities in SCZ. Mahon et al. (2015) observed greater atrophy of the left LBA and CMA in SCZ compared with bipolar disorder, while other investigations revealed reductions in the accessory basal nucleus [36,37,38]. Despite these structural findings, only a few studies have examined functional connectivity at the subregional level in SCZ [21,39]. The present work, therefore, extends prior research by characterizing both intra-amygdala connectivity and amygdala–network interactions in this disorder.

Consistent with Zhang et al. (2020), we found robust positive FC among the LBA, CMA, and SFA in both SCZ patients and controls, reflecting the tightly integrated functional organization of the amygdala. Although the overall pattern of positive intra-amygdala connectivity was preserved, the strength of specific connections differed between groups, suggesting subtle disruptions in internal amygdala communication that may contribute to SCZ symptoms [21].

Beyond intra-amygdala connectivity, we observed widespread alterations in the coupling of amygdala subregions with large-scale brain networks. Of particular note were changes involving the default mode network (DMN), a system implicated in self-referential processing, spontaneous cognition, and the generation of internally focused thought. SCZ patients showed increased FC between the left CMA and DMN regions, including the lateral parietal cortex, medial prefrontal cortex (MPFC), and posterior cingulate cortex (PCC), whereas HCs exhibited negative or weaker connections. The CMA significantly regulates attentional processing of signals in associative training and elicits emotional and physiological responses to threat or pain via projections to the brainstem, cortical, and striatal areas [49,50]. Furthermore, a recent study indicates that the main subregion impacted by the acute stressor was the CMA [51]. According to this mechanism, CMA-DMN hyperconnectivity may be exacerbated by emotional dysregulation and psychotic symptoms. These changes are consistent with a study that links emotion dysregulation to the amygdala-DMN connection [52]. Previous studies have linked DMN hyperconnectivity to negative symptoms and cognitive impairment in SCZ [53,54]. Our findings reinforce the hypothesis that aberrant DMN–amygdala interactions contribute to both cognitive deficits and core clinical symptoms.

Altered connectivity was also evident in other functional systems. Within the frontoparietal network (FPN)—a network supporting executive control—HCs demonstrated negative connections between the left amygdala subregions (LBA, CMA, SFA) and the posterior parietal cortex, a pattern absent in SCZ. Disruption of amygdala–FPN coupling may impair top-down regulation of emotion and cognition, a hallmark of the disorder. In the visual network (VN), SCZ patients exhibited increased connectivity between the left CMA and the right lateral visual cortex, as well as unique positive connections linking the amygdala (bilateral LBA, left CMA, left SFA) to visual regions. These findings align with evidence that visual processing deficits and amygdala hyperconnectivity contribute to visual hallucinations in SCZ [55].

Similarly, the sensorimotor network (SMN) showed hypoconnectivity with the right LBA in SCZ, including a loss of positive coupling with the lateral and superior SMN observed in controls. The LBA is known to integrate sensory–motor information and regulate emotional responses [56]; diminished LBA–SMN interactions may underlie motor abnormalities and thought disorder in SCZ [21]. Within the salience network (SN), positive connections between the SFA and insula, and between the CMA and supramarginal gyrus/ACC, were present in controls but absent in SCZ. Given the SN’s critical role in detecting and integrating salient stimuli, its disconnection from the amygdala could contribute to symptoms such as hallucinations, delusions, and impaired environmental monitoring [57].

Finally, SCZ patients showed aberrant amygdala coupling with language-related regions, including positive connections between the right LBA and CMA with the inferior frontal gyrus (IFG) and posterior superior temporal gyrus (pSTG). These regions are essential for speech production and comprehension, and their dysconnectivity has been linked to auditory hallucinations and disorganized thought [54]. Altered interactions were also detected with the cerebellar network (CN), where SCZ patients displayed negative connections between amygdala subregions and the posterior cerebellum, a region increasingly recognized for its role in cognition and emotion [26]. Abnormal amygdala–cerebellar coupling may contribute to the cognitive deficits commonly observed in SCZ.

Taken together, these findings highlight a pattern of hyperconnectivity with limbic and sensory networks (DMN, VN, language) and hypoconnectivity with control networks (SMN, FPN, SN) in SCZ. Such an imbalance may reflect a failure of top-down regulation of emotional and perceptual processes, resulting in positive symptoms such as hallucinations and delusions, as well as negative symptoms and cognitive impairments. The observed increase in left CMA–DMN connectivity, in particular, may represent a potential biomarker of disease severity, given its association with negative symptoms and deficits in memory and executive function.

Amygdala Subregion analyses found strong, spatially distinct changes, such as increased left CMA–DMN connectivity and altered right LBA–SMN connectivity, while the whole-amygdala analysis only found minor connectivity differences in SCZ, mainly with the visual network and a DMN node. Because divergent connection patterns may cancel out over the entire structure, this difference emphasizes how averaging across functionally varied amygdala nuclei can mask significant subregion-specific effects. Mechanistically, the LBA hypoconnectivity with sensorimotor networks may lead to changed environmental integration, whilst the CMA hyperconnectivity with DMN nodes may promote enhanced self-referential and emotional processing. These results highlight the significance of subregion-focused research in identifying the subtle changes in connections that may be responsible for the diverse symptoms seen in schizophrenia.

Several limitations must be considered. First, resting-state FC analyses remain methodologically heterogeneous, with variations in scan duration, participant eye status, and motion correction strategies potentially influencing results. Our reliance on a publicly available dataset limited control over participant selection and clinical characterization. Second, the cross-sectional design precludes conclusions about the temporal dynamics of FC changes or their relationship to illness progression. Third, the absence of diffusion tensor imaging (DTI) data limits interpretation of structural–functional relationships, and the spatial resolution of the fMRI data constrains precise delineation of amygdala subregions. Future work combining high-resolution imaging, DTI, and longitudinal designs will be critical for validating these findings and tracking FC changes over time.

## 5. Conclusions

This study demonstrates that amygdala subregions in SCZ exhibit distinct and network-specific alterations in functional connectivity, including increased coupling with the DMN and visual networks and decreased connectivity with the sensorimotor and salience networks. These abnormalities likely contribute to the diverse clinical symptoms of SCZ and may serve as potential biomarkers for early diagnosis or treatment targets. By isolating subregion-specific connectivity patterns, our results provide a more refined framework for understanding the pathophysiology of SCZ and for developing interventions aimed at restoring balanced amygdala–network interactions.

## Data Availability

Supporting this study are openly available from the UCLA Consortium for Neuropsychiatric Phenomics LA5c Study at (https://openfmri.org/dataset/ds000030/, access on 20 February 2024).
